# Application of the Partial Ozaki Procedure for Aortic Regurgitation

**DOI:** 10.1016/j.atssr.2025.08.001

**Published:** 2025-08-26

**Authors:** Masatoshi Hata, Keita Inoguchi, Junki Yokota, Noriko Kodani, Tomohiko Sakamoto, Toru Kuratani, Yoshiki Sawa

**Affiliations:** 1 Department of Cardiovascular Surgery, Osaka Keisatsu Hospital, Osaka International Medical & Science Center, Osaka, Japan

## Abstract

Aortic regurgitation (AR) arising from cusp disease poses a significant surgical challenge. In this single-center study, we evaluated a partial Ozaki technique, involving reconstruction of only the diseased cusp, and performed concomitant aortic root procedures guided by contrast-enhanced computed tomography assessment of lunule coaptation and root dimensions. Sixteen patients with severe AR who underwent partial Ozaki repair were stratified by lunule coaptation and root dimensions. No operative deaths or major complications occurred. At discharge, patients’ AR was mild. Postoperative computed tomography showed increased coaptation in the native-native and reconstructed-native groups. The partial Ozaki technique was safe and effective for cusp-insufficient AR.

Aortic regurgitation (AR) arising from cusp disease poses a unique challenge. Simple commissural resuspension often fails when the leaflet tissue is not robust enough. The Ozaki procedure—complete neocuspidization of all 3 cusps with autologous pericardium—has demonstrated excellent outcomes in treating aortic valve disease^1^^,^^2^ but may be excessive for single-cusp disease. Furthermore, dilation of the aortic root components, including the annulus, Valsalva sinuses, and sinotubular junction, contributes to AR,^3^ and lunule coaptation reflects both root morphology and repair viability.^4^^,^^5^ Achieving a lunule coaptation length of at least 6 mm is reported to be critical for durable valve repair.^4^^,^^5^ Therefore, we implemented a partial Ozaki technique, reconstructing only the diseased cusp while preserving the remaining native cusps; concomitant procedures were tailored on the basis of contrast-enhanced computed tomography (CT) assessment of the root morphology and lunule coaptation. In this report, we describe the surgical procedure of the partial Ozaki technique and present its midterm efficacy.

## Technique

From December 2022 to May 2025, 16 consecutive patients (median age, 62 years; range, 47-76 years; 12 men) with severe AR underwent partial Ozaki repair. Preoperative contrast-enhanced CT was performed in 13 patients to measure annulus, Valsalva sinus, and sinotubular junction diameters as well as lunule coaptation of intact cusps (precoaptation). Postoperative CT was performed in 5 patients to evaluate both native-native and neocusp-native coaptations.

**Figure 1:**
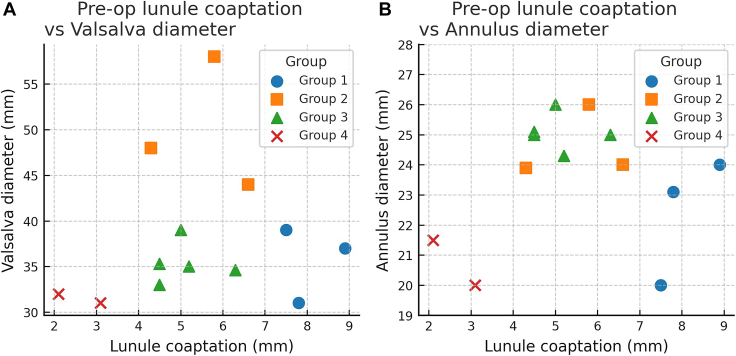
(A) Preoperative (pre-op) lunule coaptation vs Valsalva diameter (n = 13). (B) Preoperative lunule coaptation vs annulus diameter (n = 13). According to procedure, patients were assigned to 4 groups: group 1, partial Ozaki only; group 2, partial Ozaki + David root replacement; group 3, partial Ozaki + annuloplasty; group 4, conversion to full Ozaki.

Treatment decisions were stratified on the basis of preoperative coaptation and root morphology as follows. Patients with precoaptation ≥7 mm underwent partial Ozaki alone (n = 3). Those with precoaptation <7 mm and an enlarged Valsalva diameter (>40 mm) underwent partial Ozaki with a David root replacement (n = 5). Those with similar precoaptation (<7 mm) but normal root dimensions (Valsalva diameter <40 mm) underwent partial Ozaki with annuloplasty (n = 6). Finally, those with severely reduced precoaptation (<4 mm) without root enlargement required conversion to the full Ozaki procedure (n = 2) (Figure 1).

**Figure 2:**
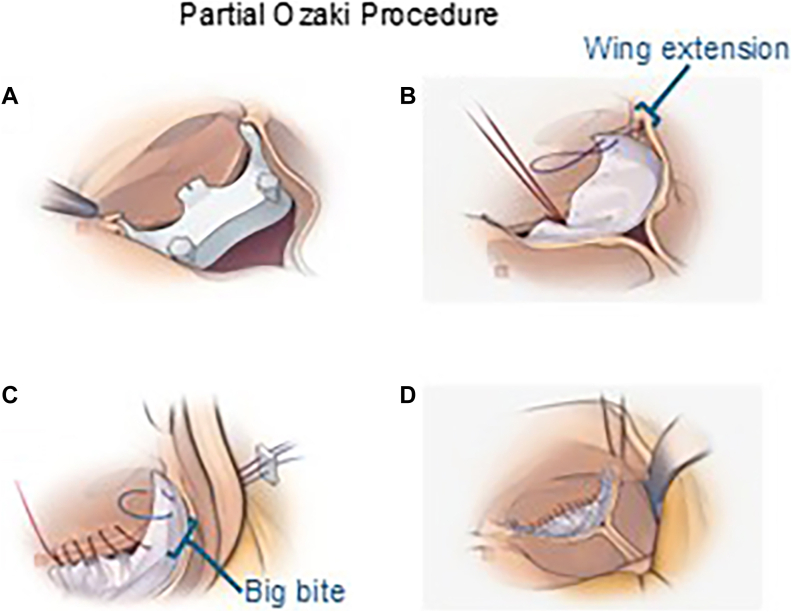
Schematic of the partial Ozaki procedure: (A) sizing; (B) anchoring with wing extension; (C) running suture; and (D) tie-down.

The surgical technique of the partial Ozaki procedure is as follows (Figure 2 and Video). After excising the diseased cusp, we measured the intercommissural distance using an Ozaki AVPro sizer (Tokyo Research Center for Advanced Surgical Technology Co Ltd). Autologous pericardium was treated with 0.6% glutaraldehyde, rinsed, and trimmed using the AVPro template. A single 4-0 suture was placed at the neocusp nadir. Two U-shaped wing-extension sutures were placed at the commissural apices, adjusting their positions to align the neocusp free edge with the tips of the adjacent native cusps. A continuous 4-0 annular suture was run from nadir to commissures, gathering tissue between nadir and big-bite. Final neocusp fixation was achieved by tying down the wing-extension sutures.

**Figure 3:**
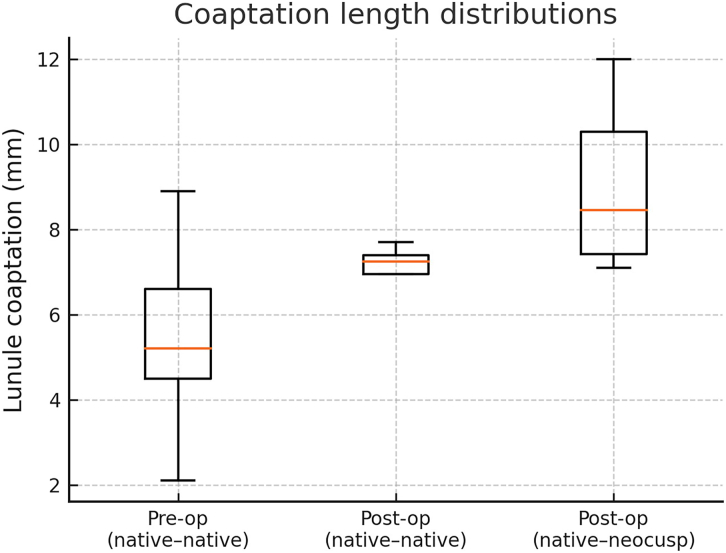
Box-and-whisker plots of computed tomography–measured coaptation in 5 patients: preoperative (pre-op) and postoperative (post-op) native intact cusps and postoperative native-neocusp.

All 16 patients survived to discharge and were alive at the last follow-up (median, 16 months; range, 6-32 months). There were no operative deaths or major complications. The median bypass time was 245 ± 65 minutes, and the median cross-clamp time was 180 ± 50 minutes. At discharge, only mild AR or less was demonstrated in all patients, with a low gradient (mean, 9.1 ± 1.4 mm Hg). Two patients had moderate AR at midterm but remained asymptomatic. The median preoperative lunule coaptation was 5.2 mm (interquartile range [IQR], 4.5-6.5 mm). Postoperative CT in 5 patients revealed native-native coaptation of 7.3 mm (IQR, 7.0-7.6 mm) and neocusp-native coaptation of 8.5 mm (IQR, 7.4-10.3 mm) (Figure 3).

## Comment

The partial Ozaki procedure is an effective cusp-sparing strategy for AR with cusp disease. However, in addition to cusp damage, root damage, including annular, Valsalva, and sinotubular junction dilation, impairs coaptation and repair durability.^3^ Preoperative CT measurement of root dimensions and lunule coaptation allows stratified decision-making. Given that a lunule coaptation length of at least 6 mm is recommended, we considered preoperative lunule coaptation of intact cusps <7 mm an indication for concomitant annuloplasty or aortic root replacement.^4^^,^^5^ Our study demonstrated that this strategy resulted in postoperative lunule coaptation >6 mm, accompanied by early valve competence and low transvalvular gradients. Integrating CT parameters into surgical planning enables tailored interventions and may improve repair outcomes.

The partial Ozaki technique is a safe and effective repair strategy for cusp-insufficient AR, provided lunule coaptation ≥6 mm is achieved directly or by adjunctive root or annuloplasty procedures. Thus, preoperative lunule coaptation measurement should be used to guide procedural planning to optimize outcomes, such as estimating the need for adjunctive root or annular procedures.
